# Reply: Specificity of RT–PCR for the detection of minimal residual disease in breast cancer patients

**DOI:** 10.1038/sj.bjc.6603130

**Published:** 2006-05-02

**Authors:** A Nissan, S Mitrani-Rosenbaum

**Affiliations:** 1 Department of Surgery and Surgical Oncology Laboratory Hadassah-Hebrew University Medical Center, Mount Scopus, Jerusalem, Israel; 2 Goldyn Savad Institute of Gene Therapy, Hadassah-Hebrew University Medical Center, Mount Scopus, Jerusalem, Israel

**Sir,**

Chechliñska *et al* raised an important issue regarding the detection of minimal residual disease in cancer patients: the presence of activated lymphoid cells in the blood and tumours of cancer patients. Despite the fact that activated lymphoid cells are present near and inside tumour tissues as well as in the blood of cancer patients, there is no published evidence that these cells express Mammaglobin A, NY-BR-1 or cytokeratin-19 (CK-19). In our study, we used 13 negative controls, peripheral blood lymphocytes from healthy donors (*n*=10) and three lymph nodes from patients with conditions other than breast cancer. All three lymph nodes were biopsied in order to rule out lymphoma and the histopatological diagnosis was reactive-inflammatory lymph nodes. None of the markers used in our study was expressed in any of the three inflammatory lymph nodes.

The high sensitivity and low specificity of the PCR detection methods may be due to contamination, – illegitimate transcription and other causes such as the presence of cells other than cancer cells expressing the same tumour antigens. However, in every biological assay, a risk of false positive results exists. The way to reduce this risk is to include more molecular markers and utilize quantitative real-time PCR that will be able to quantitate the amounts of RNA amplified. We are now in the process of analysing larger cohort of patients with more molecular markers using real-time PCR in order to correlate the presence of MRD detected by RT–PCR to patients' survival. We will adopt the idea raised by Chechliñska *et al* and will also add to our control group PBLs obtained from patients with inflammatory conditions.

